# Mosquitoborne Viruses, Czech Republic, 2002

**DOI:** 10.3201/eid1101.040444

**Published:** 2005-01

**Authors:** Zdenek Hubálek, Petr Zeman, Jiří Halouzka, Zina Juřicová, Eva Šťovíčková, Helena Bálková, Silvie Šikutová, Ivo Rudolf

**Affiliations:** * Institute of Vertebrate Biology ASCR, BrnoCzech Republic; † Health Institute, Kolín, Czech Republic; ‡ Central Bohemia Hygienic Station Prague, Mělník, Czech Republic

**Keywords:** mosquitoes, arboviruses, California group viruses, Tahyna virus, Sindbis virus, Batai virus, West Nile virus, flood, dispatch

## Abstract

Specimens from residents (n = 497) of an area affected by the 2002 flood were examined serologically for mosquitoborne viruses. Antibodies were detected against Tahyna (16%), Sindbis (1%), and Batai (0.2%) viruses, but not West Nile virus. An examination of paired serum samples showed 1 Tahyna bunyavirus (California group) infection.

The 2002 flood in Bohemia struck the Czech Republic just a few years after the 1997 flood (in Moravia and Silesia). Apart from Prague, extensive rural areas along the Vltava and Labe Rivers were flooded in August 2002. In the Mělník area, which offers favorable habitats for mosquitoes under normal conditions (*1*), mass mosquito breeding (largely *Ochlerotatus sticticus*, *Oc. cantans, Aedes vexans,* and *Ae. cinereus*) occurred after August 20. This increased mosquito population peaked September 3–9, with a biting frequency of 70 bites per person per minute. The mosquito population declined during the second half of September and disappeared by November.

## The Study

**Figure 1:**
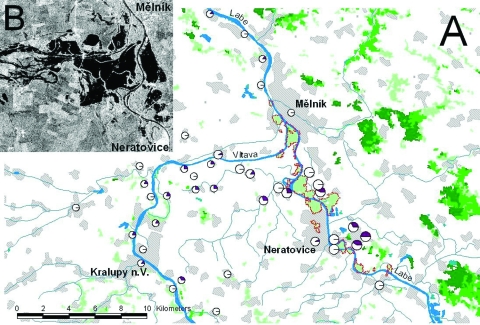
A), Potential foci of mosquitoborne viruses in the Mělník area. Floodplain forests identified on the Landsat MSS satellite images (dotted red line), with hydrology and settlement in background (DMU-200, VTOPÚ Dobruška), and proportion of Tahya virus seropositive residents at particular localities (large, medium, and small circles indicate the risk zones A, B, and C, respectively). B) [inset], radar satellite image of the conflux of the Labe and Vltava Rivers on August 17, 2002 (2 days after the flood culmination), showing extent of floodwater (dark areas). Inundated forests, with subsequent mass occurrences of *Ochlerotatus* and *Aedes* mosquitoes, are visible as lighter areas surrounding Labe River upstream of the confluence; scattered lagoons (dark areas) in arable fields along both rivers far left and right turned into breeding sites of predominantly *Culex* mosquitoes.

To estimate the risk for infections with mosquitoborne viruses, we screened the human population of the flooded area (Figure 1) for antibodies against the viruses known to occur in central Europe (*2*): Tahyna (TAHV), *Orthobunyavirus* of the California group, *Bunyaviridae*; West Nile (WNV), *Flavivirus* of the Japanese encephalitis group, *Flaviviridae*; Sindbis (SINV), *Alphavirus*, *Togaviridae*); and Batai (BATV), *Orthobunyavirus* of the Bunyamwera group, *Bunyaviridae*.

We subdivided the flooded area into 4 risk zones according to quantities of mosquitoes. Zone A was a forested floodplain along the Labe River between Obříství-Kly and Lobkovice-Kozly (11 villages), with large quantities of mosquitoes. Zone B was an intermediate area between zones A and C (5 villages, 1 small town), with fewer breeding sites but possibility for mosquito migration from zone A. Zone C was the area along the Vltava and Labe Rivers between Kralupy and Horní Počaply (25 villages and small towns), with no floodplain forests and few breeding sites for mosquitoes. Zone D was a control zone, with only sporadic occurrences of mosquitoes (mainly in Prague).

Informed written consent and serum samples were obtained from 497 survey participants of various ages from September 6 to September 13, 2002 (3 weeks after the flood culmination and 2 weeks after the mosquito emergence). Paired serum samples were taken from 150 of the survey participants 6 months later, from April 9 to May 15, 2003 (34 in zone A, 43 in zone B, 73 in zone C).

Serologic examination was performed with the hemagglutination-inhibition (HIT) and plaque-reduction neutralization tests (PRNT) in microplates (*3*–*5*). The strains used for HIT were TAHV 92, WNV Eg101, BATV 184, and SINV Eg339; a commercial control antigen (Test-Line Ltd., Brno, Czech Republic) of Central European tickborne encephalitis virus (CEEV) was used. All serum samples were acetone-extracted and tested with sucrose- and acetone-processed antigens by using 8 hemagglutinin units; titers >20 were considered positive. For PRNT, TAHV T16, WNV Eg101, CEEV Hypr, and BATV, 184 viral strains were used. The test was conducted on Vero or SPEV (embryonic pig kidney: for CEEV) cells. All serum samples were heat inactivated and screened for antibodies at 1:8; those reducing the number of virus plaques by 90% were considered positive and titrated to estimate dilutions causing plaque–number reduction by 50% (PRNT_50_) and 90% (PRNT_90_). The serum samples reacting with WNV were examined for cross-reactivity with CEEV. PRNT with BATV was used only as a confirmatory test for the serum samples reacting with BATV in HIT.

**Figure 2:**
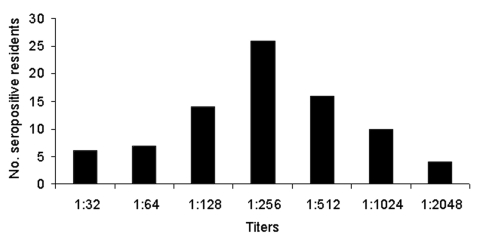
Distribution of 50% plaque-reduction neutralization titers of antibodies to Tahyna virus (y axis, number of seroreactors).

Against TAHV, 82 (16.5%) of 497 study participants had neutralizing antibodies, and 74 (14.9%) were seropositive in HIT. In PRNT_50_, the titers were 32–2048 (geometric mean titer [GMT] 260), in PRNT_90_ 16–1024 (GMT 119), and in HIT 20–40 to 160 (GMT 40). Figure 2 illustrates the distribution of neutralizing antibody titers. No difference occurred in neutralizing antibody prevalence between sexes, 32 (15.8%) of 202 males and 50 (16.9%) of 295 females (χ^2^ = 0.11; p = 0.744). The prevalence rate increased significantly with age (Table 1: χ^2^ = 39,809; p <0.001); TAHV antibodies were found infrequently in persons <19 years of age. Neutralizing antibody distribution, with respect to the residence location (Table 2, Figure 1), showed the highest seroprevalence in zone A (28%), lower seroprevalences in zones B and C, and 5% in the control zone D (χ^2^ = 14.57; p = 0.002). Significant differences were found between zone D and all other zones, and between zones A and C (χ^2^ = 7.243; p = 0.007), but not between zones A and B or B and C; HIT yielded similar results. The seroprevalence in relation to the proximity of study participants’ locations to the nearest floodplain forest within zones A, B, and C demonstrated decreasing seroprevalence with increasing proximity to the forest (χ^2^ = 8.51; p = 0.003) (Table 2).

**Table 1:** Comparison of the prevalence of neutralizing antibodies to Tahyna virus by age groups after the floods in central Bohemia in 2002 and southern Moravia in 1997*†

Age (y)	CB 2002, n	% positive	SM 1997, n	% positive
0–9	18	5.6	39	0.0
10–19	53	0.0	49	8.2
20–29	74	5.4	128	19.5
30–39	69	17.4	79	63.3
40–49	62	11.3	80	62.5
50–59	86	19.8	90	81.1
60–69	78	32.1	59	79.7
>70	57	28.1	95	88.4

*CB, central Bohemia; SM, southern Moravia; n, number of residents examined. †Source (6).

**Table 2:** Prevalence of neutralizing antibodies to Tahyna virus after the 2002 flood, Central Bohemia*

	n†	% positive
Risk zone		
A	75	28.0
B	83	20.5
C	279	14.7
D	60	5.0
Distance to FPF (km)		
<1.0	78	28.2
1.0–2.9	75	21.3
3.0–5.9	70	17.1
≥6.0	214	13.6

*As related to the residence location: risk zones A to D; and distance to floodplain forest (FPF, within zones A, B, and C only). †n, number of residents examined.

Against WNV, no specific reactions were found. Although serum samples from 34 (6.8%) study participants reacted in HIT with the WNV at titers 40 to 80, all of them also reacted with CEEV at titers similar or higher (≤160). CEEV could have occurred in the area, and some study participants may have been vaccinated against tickborne encephalitis. In PRNT_90_, 6 study participants (1.2%) reacted with WNV but at low titers of 8 to 16; these serum samples also reacted in PRNT with CEEV; thus, the results were considered to be crossreactions as well. Additionally, 42 (8.5%) seroreactors against WNV appeared in the less stringent PRNT_50_, but all titers were low (8–32) and cross-reacted with CEEV.

Against SINV, antibodies were tested with HIT only and detected in specimens from 7 (1.4%) study participants, with low titers of 20 to 40. Of the BATV, specimens from 7 study participants reacted in HIT at a low titer of 20. By confirmatory testing of these serum samples in PRNT, only 1 (0.2%) showed specific antibodies to BATV; the titer was 64 in PRNT_50_ and 32 in PRNT_90_.

Seroconversion (>4-fold rise in titer) was found with TAHV only. After the flood the infection episode occurred in one 55-year-old woman from Obříství (zone A), as shown by the seroconversion in both HIT (<20/40) and PRNT_50_ (<8/512). Three other study participants seroconverted in 1 test only: a 40-year-old man from Chlumín, zone B (HIT 20/80; PRNT 128/128); a 32-year-old man from Chlumín (HIT <20/20–40; PRNT 128/64); and an 80-year-old woman from Obříství (HIT 20/80; PRNT 64/32). These results are less convincing. Upon our request, local general practitioners did not corroborate consistent signs of a disease reported by these 4 study participants from October 2002 to April 2003. In general, clinical symptoms of TAHV infection are milder in adults than in children (*7*). Seroconversion against mosquitoborne viruses was not detected in any of the 73 study participants in zone C.

## Conclusions

On the basis of this serosurvey, recent infections with WNV (in contrast to South Moravia after the 1997 flood (*5*,*6*), SINV, and BATV have not been found in Central Bohemia after the flood. However, activity of another mosquitoborne virus, TAHV, has been found in a natural focus along the Labe River at Neratovice. This focus has so far gone unnoticed (*8*). Lower frequency of TAHV antibodies has been detected along the lower reaches of the Vltava River. The prevalence of antibodies to TAHV increased with risk-zone ranking (from zone D to the highest risk zone A) and with decreasing distance to floodplain forests, the primary breeding habitat of vector mosquitoes (*9*–*11*).

In disease-endemic areas, the proportion of residents with antibodies against California group viruses increase with age (*6*,*12*). A similar situation occurred in the Central Bohemian flooded area, where antibodies to TAHV were detected in a low proportion of residents <20 years of age. Nevertheless, TAHV seems to be active in the area. At least 1 seroconversion among 150 residents (attack rate ≈0.67%) against TAHV has been proven. With ≈100,000 inhabitants in the risk zones (1992 census), ≈670 (95% confidence interval 20–3,719) persons could have been infected after the flood.

Environmental factors, such as heavy rains followed by a flood, artificial inundation of floodplain forests, or rehabilitation of wetlands that support mosquito-vector populations, could give rise to preconditions for an increased incidence of mosquitoborne infectious diseases, even in temperate climates. Under such circumstances, the optimum strategy is an epidemiologic surveillance that includes monitoring, especially of infection rate of mosquito populations and incidence of mosquitoborne diseases in humans. The surveillance results could then be used in integrated mosquito control.
